# Effects of Age-Related Stereotype Threat on Metacognition

**DOI:** 10.3389/fpsyg.2020.604978

**Published:** 2020-12-02

**Authors:** Natasha Y. Fourquet, Tara K. Patterson, Changrui Li, Alan D. Castel, Barbara J. Knowlton

**Affiliations:** ^1^ Department of Psychology, Northern Virginia Community College, Sterling, VA, United States; ^2^ Department of Psychology, University of California, Los Angeles, Los Angeles, CA, United States; ^3^ Department of Psychology, Georgia State University, Atlanta, GA, United States

**Keywords:** aging, memory, metamemory, stereotype threat, value

## Abstract

Previous work has shown that memory performance in older adults is affected by activation of a stereotype of age-related memory decline. In the present experiment, we examined whether stereotype threat would affect metamemory in older adults; that is, whether under stereotype threat they make poorer judgments about what they could remember. We tested older adults (*M*_Age_ = 66.18 years) on a task in which participants viewed words paired with point values and “bet” on whether they could later recall each word. If they bet on and recalled a word, they gained those points, but if they bet on and failed to recall a word, they lost those points. Thus, this task required participants to monitor how much they could remember and prioritize high value items. Participants performed this task over six lists of items either under stereotype threat about age-related memory decline or not under stereotype threat. Participants from both groups performed similarly on initial lists, but on later lists, participants under stereotype threat showed impaired performance as indicated by a lower average point score and a lower average gamma coefficient. The results suggest that a modest effect of stereotype threat on recall combined with a modest effect on metacognitive judgments to result in a performance deficit. This pattern of results may reflect an effect of stereotype threat on executive control reducing the ability to strategically use memory.

## Introduction

Older adults have been shown to exhibit deficits in explicit memory and executive function compared to younger adults (see Luo and Craik, 2008; Nyberg et al., 2012, for reviews). There are both biological and contextual causes that may lead to cognitive performance decline in older adults. One contextual factor that may impact cognitive performance in older adults is exposure to negative stereotypes about aging. Stereotype threat arises when a person is concerned that their performance will confirm a negative stereotype about their group. The seminal study by Steele and Aronson (1995) had a sample comprised of African American and Caucasian participants, who were asked to answer questions taken from the Graduate Record Examination’s (GRE) verbal section. Half of the participants were told that the questions assessed verbal ability (i.e., diagnostic condition). For the remaining half, the instructions did not make any reference to verbal ability; rather, the experimenters explained that they were interested in examining psychological factors affecting verbal problem-solving (i.e., non-diagnostic condition). The data showed that when the test was presented as diagnostic, African American participants performed worse than Caucasian peers and African Americans in the non-diagnostic condition.

Subsequent studies have documented similar stereotype threat effects in other groups (Aronson et al., 1999; Spencer et al., 1999; Stone et al., 1999; Gonzales et al., 2002). In older adults, activation of the widely-held negative stereotype that old age is associated with forgetfulness has been shown to reduce memory performance (for a review, see Barber and Mather, 2014). In some experiments, stereotype threat is induced by exposing the participant to materials that explicitly state negative age-related stereotypes. For example, Hess et al. (2003) had participants read a text passage stating that older adults may have to increasingly depend on the help of memory tools, family, and friends to cope with age-related memory decline. Other methods used to induce age-based stereotype threat include telling participants the goal of the experiment is to examine age-related differences in performance (e.g., Hess et al., 2009), labeling the task as one that evaluates memory (e.g., Desrichard and Köpetz, 2005), asking participants to report their age (e.g., Kang and Chasteen, 2009), and implicitly presenting words related to the stereotype (e.g., Hess et al., 2004).

Although the effects of stereotype threat have been widely documented, less is known about the cognitive mechanisms through which stereotype threat affects older adults. Two leading theories on the mechanisms of stereotype threat are that stereotype threat taxes executive control resources (Schmader and Johns, 2003; Schmader et al., 2008) and that stereotype threat creates an imbalance in regulatory fit (Seibt and Förster, 2004; Grimm et al., 2009). According to the executive function view, stereotype threat affects performance through three distinct, yet interconnected cognitive processes (Schmader et al., 2008). First, stereotype threat induces a state of divided attention, which makes it harder for participants to manage the task at hand. Second, stereotype threat induces stress. Stress affects neural activity in prefrontal areas (e.g., Arnsten, 2009; Schoofs et al., 2009), which play a major role in executive function. Lastly, stereotype threat induces negative mood, thus requiring emotion regulation. Divided attention, stress, and negative mood converge to tax executive resources, which are essential for any cognitive task.

The research on the effects of stereotype threat on executive function in older adults is mixed. Hess et al. (2009) employed a computation-span task to assess working memory – an important component of executive function. Results showed that there were no significant differences in working memory performance between older adults in the stereotype threat condition and older adults in the non-threat condition. Given this finding, Hess et al. argued that working memory may not be susceptible to threat. However, this lack of statistical significance may be due to the way the computation-span task was presented to the participants. Rather than being presented as a memory task, it was framed as a measure of quantitative skills, which may not be an effective label to induce threat in older adults. Other studies have found differences in executive function performance between older adults under stereotype threat and in non-threat conditions. Mazerolle et al. (2012) used a reading span task to assess working memory and found that older adults under threat had poorer performance than both young adults and older adults in the control condition. Mazerolle et al. (2012) also examined controlled vs. automatic responses using a word stem completion task and found that older adults under threat displayed more automatic responses than controlled responses, compared to older adults in the non-threat condition. Taken together, these findings provide support for the idea that, under specific experimental conditions, executive function may in fact be affected when older adults face stereotype threat.

Additional studies have found counterevidence for the executive function hypothesis, and support an alternative one, the regulatory fit hypothesis. This hypothesis stems from the idea that individuals differ in their approach to accomplishing goals (Higgins, 1997). On one hand, some people have a promotion focus, which prioritizes gains. On the other hand, some people have a prevention focus, which underscores an absence of losses. Research has found that performance can be maximized when there is a regulatory fit, that is, when the reward structure is in tune with the regulatory focus of the individual (Higgins, 2000). Regulatory focus can be manipulated experimentally for a short period of time. Stereotype threat has been proposed to induce a prevention focus state, leading individuals to prevent losses rather than to maximize their gains (e.g., Seibt and Förster, 2004). According to the regulatory fit hypothesis of stereotype threat, this prevention focus will result in impaired performance when gains are emphasized but enhanced performance when losses are emphasized.

Barber and Mather (2013a) tested this idea with an older adult sample using a sentence span task and a reward structure (i.e., gain‐ or loss-based) manipulation. In the gain-based condition, participants received two poker chips whenever they recalled a word. In the loss-based condition, three poker chips were subtracted from a total of 100 chips for every word that participants failed to recall. In the gain-based structure, stereotype threat negatively affected the number of words recalled. However, the number of words recalled increased significantly under stereotype threat in the loss-based structure. In another study by Barber and Mather (2013b), older adults under stereotype threat made fewer memory errors (i.e., intrusions and false alarms) compared to older adults in the control condition. Along these lines, Popham and Hess (2015) manipulated stereotype threat while older adults performed a letter-canceling task. The results showed that participants in the threat condition were slower, but more accurate, than those in the no-threat condition. Together, these findings show that executive control resource depletion may not fully explain the decrement in cognitive performance found in older adults who are under threat, given that in some cases, stereotype threat actually enhances performance.

As highlighted by the research presented above, there may be several plausible cognitive mechanisms through which stereotype threat affects cognitive performance in older adults. In the present study, we used a value-directed remembering task to assess memory selectivity and metamemory – abilities that have been shown to be generally spared in normal aging (e.g., Castel et al., 2002, 2009). The goal of this study was to assess whether stereotype threat (a) affects performance in tasks where older adults have been proven to excel and (b) how stereotype threat impacts the way older adults think about their own memory.

In the value-directed remembering task (Castel et al., 2002), participants are presented words that are paired with arbitrary point values that indicate the value of each word. Participants are instructed that the objective of this task is to maximize the amount of points they earn for remembering the words. Words and point values are presented in a list format on a computer screen. After each list is presented, participants are prompted to recall all the words (not the point values) that they are able to recall. Not surprisingly, results have shown that older adults recall fewer words than younger adults. However, older adults have spared ability to focus their memory resources on words with high point values, enabling them to maximize the amount of points they are able to earn (e.g., Castel et al., 2002, 2009). These findings highlight that while older adults are impaired in terms of the quantity of information they can retain, they can still recall high-value information and be selective with regards to the information they attend to.

The current study used a value-directed paradigm as modified by McGillivray and Castel (2011) that allowed us to assess how older adults make metacognitive judgments in the context of stereotype threat. In this version of the task, participants “gamble” on their ability to remember high-value information. As in the original task (Castel et al., 2002), participants are presented with words and point value pairings across multiple study-test cycles. For each word presented, participants have to choose if they want to “bet” on the word. If they successfully recall “bet on” words, participants are awarded the amount of points that the word is worth but lose the points if they do not recall the word. Participants are informed that the task’s goal is to get as many points as possible, and are encouraged to try to maximize gains and minimize losses.

McGillivray and Castel (2011) tested this task with a sample of young and older adults. Results showed a similar pattern to the original value-directed paradigms – although older adults were not able to recall the same number of words as young adults, they were able to obtain a similar amount of points with increasing task experience. McGillivray and Castel found that older adults (and young adults) were initially overly confident, betting on more words than they could recall. But, as the task progressed, older adults were able to implement more effective strategies. For example, older adults showed greatly improved calibration score (i.e., “bet on” words vs. remembered words). Given that older adults improved their calibration score with task experience, these findings suggested that – to some extent – people show preserved metacognitive judgments about their memory capacity as they age.

To our knowledge, the current study is the first to look at how older adults selectively remember important information and how they judge their memory abilities when faced with stereotype threat. If stereotype threat induces a prevention focus, older adults in the stereotype threat condition might try to minimize their losses during the task rather than focusing on gains. If this is the case, we would expect to see lower levels of betting in participants in the stereotype threat condition relative to those in the control condition, which might result in fewer points gained but also fewer points lost. A focus on minimizing losses might also result in better calibration scores in the stereotype threat group, especially toward the beginning of the task when people who are not under stereotype threat tend to show overconfidence. On the other hand, if stereotype threat impairs executive function, older adults under stereotype threat might show diminished ability to adjust their betting behavior with task experience, resulting in metamemory impairment on later lists in the stereotype threat group relative to the control group.

We also recruited participants from a wide age range in order to investigate the effects of age on task performance. Previous research has shown that older adults on the younger end of the age spectrum show more pronounced stereotype threat effects than older adults on the older end of the age spectrum (Hess et al., 2009; Eich et al., 2014). These findings suggest that transitioning from middle to older age may be a particularly vulnerable time for stereotype threat. Therefore, we predicted that the effects of stereotype threat on metacognition might be larger in older adults on the younger end of the age spectrum.

## Materials and Methods

### Participants

Older adults (*N* = 44; 22 women) were recruited for the current study. Participants were recruited through a newspaper advertisement. The average age was 66.18 years (*SD* = 10.85, range = 50–88). Participants had completed an average of 15.75 years of education (*SD* = 1.45, range = 12–17) and had an average Mini Mental Status Exam (MMSE) score of 28.95 (*SD* = 1.03, range = 26–30; maximum score = 30; Folstein et al., 1975). Seventeen participants were ages 50–59, 11 were ages 60–69, and 16 were ages 70 and up (see Table 1 for sample characteristics by age category).

**Table 1:** Sample characteristics by age category.

Age category	50–59 (*N* = 17)	60–69 (*N* = 11)	70+ (*N* = 16)
Age (years)	55.88 (2.96)	64.09 (3.56)	78.56 (5.75)
Gender (% female)	58.82	45.45	43.75
Education (years)	14.88 (1.65)	16.45 (0.52)	16.19 (1.22)
MMSE score	29.35 (0.86)	28.64 (0.92)	28.75 (1.18)

Mean (*SD*) demographics for participants aged 50–59, 60–69, and 70+. MMSE, Mini Mental Status Exam (Folstein et al., 1975).

Half of the sample was randomly assigned to a neutral condition and the other half to a stereotype threat condition. Participants were tested in the Cognitive Neuroscience Laboratory at the University of California, Los Angeles. Participants’ parking expenses were covered and they received $20 as compensation. Study procedures were approved by the Institutional Review Board of the University of California, Los Angeles, and all participants provided written record of informed consent.

Sample size was determined based on our previous study in which significant effects of age-related stereotype threat were found in recall performance in adults aged 53–74 using a sample of 42 participants (Eich et al., 2014). The slightly larger sample size in the present study allows detection of effects of size *d* = 0.86 in two-tailed comparisons with a 0.8 probability.

### Design

We used a 3 × 2 mixed-subjects design that enabled us to examine effects of stereotype threat and changes in performance with practice. The first independent variable was List. Participants saw a total of six lists; however, lists were collapsed into beginning, middle, and end to maintain statistical power. That is, Lists 1 and 2 were averaged into a single measure (i.e., “beginning”). The same was done with Lists 3 and 4 (i.e., “middle”) and Lists 5 and 6 (i.e., “end”). The second independent variable was Group. Participants were randomly assigned to either a Neutral or Stereotype Threat condition.

### Materials

#### Gambling Task

Seventy-two common nouns were used as stimuli. The nouns were presented for 5 s across six lists with 12 words each. All words contained four to five letters. Each word was randomly assigned a point value. Point values ranged from 1–10, 15, and 20. Each point value was only used once within a list, and the order of the point values within and across lists varied (e.g., the seven-point word may appear first on List 1 but fifth on List 2). Participants had 90 s to type all the words that they could recall immediately after the last word was presented.

#### Stereotype Threat Manipulation

We constructed two paragraphs for participants to read which were adapted from the manipulation used by Hess et al. (2003; see Appendix). The two paragraphs differed in content, which was either neutral or threat related. The neutral paragraph served as our control manipulation and depicted a non-biased view of aging, which did not mention possibly stereotyped words such as memory or memory deterioration. This paragraph stated that participants would take part in a *cognitive* task. The threat manipulation emphasized a negative view of aging. The threat paragraph highlighted words like *memory* and the fact that it *deteriorates* with age. This paragraph stated that participants would take part in a *memory* task. Moreover, the text in the neutral paragraph was followed by a prompt for participants to provide their subject ID. The threat paragraph prompted participants for their age, which was meant to be a reminder of their age category.

### Procedure

Participants first gave informed consent for their participation in the study. Next, they received instructions to complete the gambling task. The instructions explained that they would see words paired with point values to indicate each word’s worth. Participants were also informed that they would be making bets on a trial by trial basis. The scoring schema was also delineated: (a) They would earn points whenever they bet on a word and later recalled it and (b) lose the points if they failed to recall a word that they had placed a bet on. Participants were given the following instruction regarding their task goal: “It is up to you how you will gamble/bet on the words, but your goal is to get as many points as possible (thus, you want to maximize your gains but minimize any losses).” Participants were then given the chance to ask any questions before beginning the task, and then they were instructed to read the paragraph corresponding to their assigned condition. The words were displayed on the computer screen for 5 s, and participants had to indicate if they wanted to bet on each word by clicking “yes,” or “no” if they did not want to bet. If no choice was made it was calculated as a “no.” After a list of 12 words was shown, participants were prompted with blanks where they would type in the words they remembered. Participants had a 90 s recall period and were given their total score after each list. This study-test cycle repeated for a total of six lists. After completing the gambling task, participants completed demographic questionnaires and the MMSE. They were compensated for their participation and those in the stereotype threat condition were debriefed at the end of the session.

## Results

### Total Score

**Figure 1:**
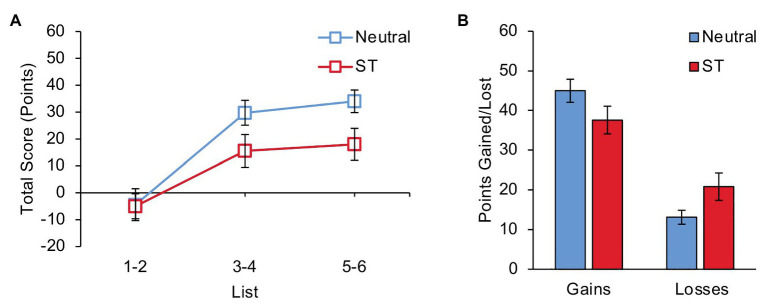
**(A)** Average total score (points gained minus points lost) on lists 1–2, lists 3–4, and lists 5–6 for participants in the Neutral and Stereotype Threat groups. **(B)** Average points gained and points lost on lists 3–6 for participants in the Neutral and Stereotype Threat groups. ST, Stereotype Threat. Error bars represent standard error of the mean.

During this task, participants were instructed that their goal was to get as high a score as possible, and were given feedback on their score after each list. This total score, calculated as the points gained from successfully recalling words on which bets were placed minus the points lost from failing to recall words on which bets were placed, is a measure of the participant’s overall performance on the task. The average total score obtained by each group on lists 1–2, lists 3–4, and lists 5–6 is shown in Figure 1A. A 3 (List: Beginning, Middle, and End) × 2 (Group: Neutral and Stereotype Threat) ANOVA revealed a main effect of List, *F*(2, 84) = 43.38, *p* < 0.001, *η*_p_^2^ = 0.508, and a marginal List × Group interaction, *F*(2, 84) = 2.72, *p* = 0.072, *η*_p_^2^ = 0.061. As can be seen in the figure, the average total score increased from the beginning to the middle of the experiment, *t*(43) = 7.17, *p* < 0.001, *d* = 1.08, and then remained at a similar level from the middle to the end of the experiment, *t*(43) = 1.03, *p* = 0.307, *d* = 0.16. During lists 1–2, the Neutral group and the Stereotype Threat group obtained similar average total scores, *t*(42) = 0.08, *p* = 0.938, *d* = 0.02. During lists 3–6, the average total score obtained by the Neutral group was twice as high as the average total score obtained by the Stereotype Threat group, *t*(42) = 2.25, *p* = 0.030, *d* = 0.68. These results suggest that although participants in both groups performed poorly on the initial lists, losing on average more points than they gained, the Neutral group was able to modify their initial strategy more effectively to achieve better performance on the final four lists.

### Gains and Losses

To further investigate the effect of stereotype threat on total score during the final four lists, we looked at the effects of group on points gained and points lost separately. Successful performance on this task requires both maximization of gains and minimization of losses. The average points gained and lost by each group during lists 3–6 is shown in Figure 1B. Compared to the Neutral group, the Stereotype Threat group gained numerically fewer points from successful bets and lost numerically more points from unsuccessful bets, but neither of these differences were statistically significant, although there was a trend for more losses from unsuccessful bets in the stereotype threat condition [*t*(42) = 1.62, *p* = 0.112, *d* = 0.49 (gains), *t*(42) = 1.99, *p* = 0.054, *d* = 0.60 (losses)]. Therefore, it appears that modest differences in both gains and losses contributed to the observed group difference in total score during the final four lists.

### Calibration Score

**Figure 2:**
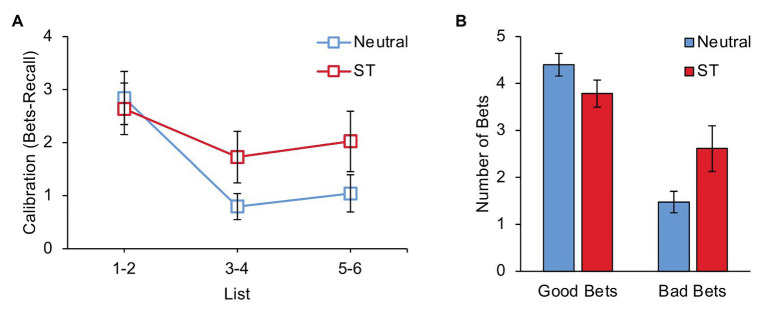
**(A)** Average calibration score (number of items bet on minus number of items recalled) on lists 1–2, lists 3–4, and lists 5–6 for participants in the Neutral and Stereotype Threat groups. **(B)** Average number of successful (“good”) and unsuccessful (“bad”) bets during lists 3–6 for participants in the Neutral and Stereotype Threat groups. ST, Stereotype Threat. Error bars represent standard error of the mean.

Successful performance on this task requires that participants calibrate their betting behavior to their recall ability. To examine this, we calculated participants’ calibration score for each list, computed as the number of items on which bets were placed minus the number of items actually recalled. The ideal calibration score is zero, meaning the participant bet on exactly as many items as they were able to recall. The average calibration score obtained by each group on lists 1–2, lists 3–4, and lists 5–6 is shown in Figure 2A. A 3 (List: Beginning, Middle, and End) × 2 (Group: Neutral and Stereotype Threat) ANOVA revealed a main effect of List, *F*(2, 84) = 11.29, *p* < 0.001, *η*_p_^2^ = 0.212. The List × Group interaction was not significant, *F*(2, 84) = 2.05, *p* = 0.135, *η*_p_^2^ = 0.047. As can be seen in the figure, participants’ calibration improved from the beginning to the middle of the experiment, *t*(43) = 4.26, *p* < 0.001, *d* = 0.64, and then remained at a similar level from the middle to the end of the experiment, *t*(43) = 1.04, *p* = 0.306, *d* = 0.16. During lists 1–2, the Neutral group and the Stereotype Threat group obtained similar average calibration scores, *t*(42) = 0.29, *p* = 0.771, *d* = 0.09. During lists 3–6, the average calibration score obtained by the Neutral group was marginally better than the average calibration score obtained by the Stereotype Threat group, *t*(42) = 1.75, *p* = 0.088, *d* = 0.53.

### Bets and Recall

We next investigated the effect of stereotype threat on betting and recall during lists 3–6. Compared to the Neutral group, the Stereotype Threat group placed numerically more bets and recalled numerically fewer items, but neither of these differences were significant, *t*(42) = 0.80, *p* = 0.431, *d* = 0.24 (bets), *t*(42) = 1.21, *p* = 0.231, *d* = 0.37 (recall). An analysis of bets based on whether the bet was a “good bet” (i.e., the item was subsequently recalled) or a “bad bet” (i.e., the item was subsequently not recalled) revealed a Bet Success × Group interaction, *F*(1, 42) = 7.25, *p* = 0.010, *η*_p_^2^ = 0.147 (Figure 2B). Compared to the Neutral group, the Stereotype Threat group placed numerically fewer good bets, but this difference was not statistically significant, *t*(42) = 1.63, *p* = 0.110, *d* = 0.49. In contrast, there was a significant difference in the number of bad bets, such that the Stereotype Threat group placed more bad bets than the Neutral group, *t*(42) = 2.13, *p* = 0.039, *d* = 0.64.

### Gamma Coefficient

**Figure 3:**
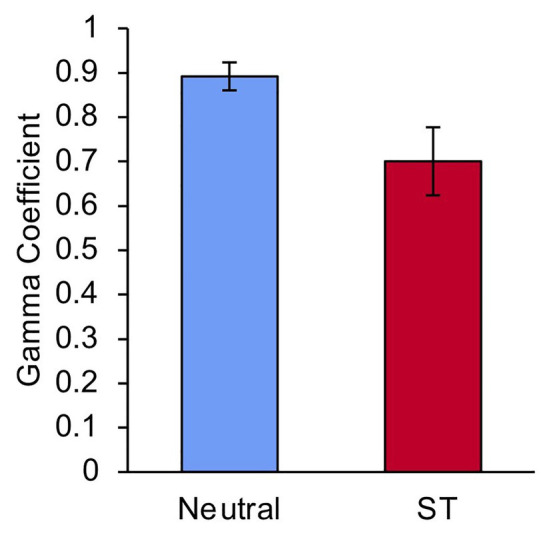
Average gamma coefficient on lists 3–6 for participants in the Neutral and Stereotype Threat groups. ST, Stereotype Threat. Error bars represent standard error of the mean.

To further examine the relationship between participants’ betting and recall, we calculated the gamma coefficient (Goodman and Kruskal, 1954) for each participant across lists 3–6. Gamma is a commonly used measure of metacognitive ability that looks at the correspondence between participants’ predictions of their cognitive ability (in this case, betting) and their subsequent performance (in this case, recall). Gamma ranges from −1 to +1, with +1 reflecting stronger association between predicted performance and actual performance. One participant from the Stereotype Threat group was excluded from this analysis because a gamma coefficient could not be calculated due to invariance in betting. The average gamma coefficient for each group during lists 3–6 is shown in Figure 3. Compared to the Neutral group, the Stereotype Threat group had a significantly lower gamma coefficient, *t*(41) = 2.35, *p* = 0.024, *d* = 0.72, indicating a diminished ability to judge which items they would be able to recall.

### Effects of Age

To test for the effects of age on performance of this task, we conducted a series of linear regression analyses that included Age as a continuous predictor variable, Group as a dummy-coded dichotomous predictor variable (Neutral = 0, Stereotype Threat = 1), and the interaction of Age and Group. Outcome variables were calculated across lists 3–6. The results of this set of analyses are shown in Table 2. Age was a significantly negative predictor of recall (*B* = −0.05, *p* = 0.023), bets (*B* = −0.11, *p* = 0.010), and good bets (*B* = −0.07, *p* = 0.004). There were no significant Age × Group interactions (smallest *p* = 0.162), indicating that the impact of stereotype threat did not vary with age.

**Table 2:** Summary of linear regression analyses of age effects on outcome variables during lists 3–6.

Outcome	Predictor	*B*	*SE*
Total score	Age	−0.17	0.50
	Group	−31.88	43.88
	Age × Group	0.26	0.66
Gains	Age	−0.37	0.33
	Group	−9.50	28.84
	Age × Group	0.05	0.43
Losses	Age	−0.20	0.28
	Group	22.38	24.43
	Age × Group	−0.21	0.37
Calibration	Age	−0.05	0.04
	Group	2.42	3.30
	Age × Group	−0.02	0.05
Bets	Age	−0.11^**^	0.04
	Group	2.31	3.47
	Age × Group	−0.02	0.05
Recall	Age	−0.05^*^	0.02
	Group	−0.11	2.01
	Age × Group	0.00	0.03
Good bets	Age	−0.07^**^	0.02
	Group	−1.71	2.07
	Age × Group	0.02	0.03
Bad bets	Age	−0.03	0.04
	Group	4.01	3.25
	Age × Group	−0.04	0.05
Gamma	Age	0.00	0.01
	Group	−0.94^†^	0.52
	Age × Group	0.01	0.01

† *p* < 0.10;

* *p* < 0.05;

** *p* < 0.01.

## Discussion

The objective of the current study was to examine the effect of stereotype threat on metacognition. We used a gambling version of the value-directed remembering task, which allowed us to study how older adults prioritize information and how they assess their own memory abilities while under stereotype threat. Previous research conducted by McGillivray and Castel (2011) has documented that older adults performing this task remember fewer words than younger controls, but that older adults still obtain a comparable amount of points in later lists. McGillivray and Castel found that older adults were initially overconfident, exemplified by their excessive betting on earlier lists but managed to calibrate bets and recall more successfully in later lists.

In the current study, both the Neutral and Stereotype Threat groups initially misjudged their memory capacity, losing more points than they were able to earn and betting on more words than they were able to recall. McGillivray and Castel (2011) found that task experience helped older adults’ initial “metacognitive failure,” and our data from the Neutral group replicate these findings. In our study, participants in the Neutral condition benefitted from task experience, which supports the idea that older adults have the ability to incorporate metacognitive knowledge when navigating memory tasks. This finding is consistent with previous research showing that older adults’ metamemory is comparable to that of young adults (e.g., Hertzog and Hultsch 2000; Souchay and Isingrini, 2004; McGillivray and Castel, 2011). However, our data from the Stereotype Threat group suggest that they may not benefit as much from task experience, resulting in impairment on later lists relative to the Neutral group. It is possible that stereotype threat overrides the benefit of task experience in this metacognitive task. Older adults may be preoccupied with the stereotype of poor memory in aging; consequently, cognitive resources devoted to adapting an effective strategy may become compromised. Previous research has shown that strategy is affected when older adults navigate a memory task under threat. For example, Hess and Hinson (2006) showed that older adults under threat underutilized clustering (i.e., grouping words of a similar semantic category). Strategy is crucial to maximize the overall score; therefore, metacognition became highly important in the current study.

Metacognition and executive function both require control and monitoring information in order to execute a desired voluntary action. Previous research has found that older adults who show greater executive control also display better metacognition (Souchay and Isingrini, 2004). The frontal cortex also plays a role in metacognitive monitoring (Fernandez-Duque et al., 2000). For instance, patients with frontal lobe damage display deficits in metacognition (e.g., Shimamura and Squire, 1986; Janowsky et al., 1989). The evidence of the role of frontal areas in executive control is widely supported. Taken together, these findings suggest a strong overlap in cognitive processes and a convergence of brain regions that subserve metacognition and executive control. The pattern of results observed in our study appears to be consistent with the idea that stereotype threat in older adults can impair executive function (e.g., Mazerolle et al., 2012) and that this impairment can exacerbate memory deficits. Specifically, we found that in later lists, the Stereotype Threat group obtained significantly lower total scores and marginally worse calibration scores than the Neutral group. Both of these measures require executive function to monitor performance, strategically decide which items to bet on, and adjust strategy to improve scores. Impaired metacognition was also demonstrated by a significantly lower average gamma coefficient during later lists in the Stereotype Threat group, indicating a diminished ability to judge which items they would be able to recall.

Supporters of an alternative hypothesis of stereotype threat, the regulatory fit hypothesis, have argued that stereotype threat induces a prevention focus (i.e., instead of a promotion focus; Seibt and Förster, 2004), which makes individuals under threat become more concerned with not doing their worst and less worried about doing their best. Consistent with this perspective, research has found that participants under threat recall fewer words but make fewer errors (e.g., Barber and Mather, 2013b). One way we measured errors in the current study was by quantifying points lost when words that were bet on were later forgotten during the free recall portion of the task. We found that older adults in the Stereotype Threat group lost marginally more points in later lists compared to the those in the Neutral group, which appears to be inconsistent with a focus on loss prevention. Similarly, we predicted that a focus on loss prevention might result in lower levels of betting in the Stereotype Threat group, but this is not what we found – in fact, the Stereotype Threat group placed numerically more bets. Moreover, we found that the Stereotype Threat group placed significantly more “bad bets” (bets on items that were not subsequently recalled) in later lists compared to the Neutral group, indicating that they did not learn to stop making bad bets like participants in the Neutral group did. In the current study, participants had to consider both the point value of each item as well as their judgment of learning the item in order to bet effectively. Participants also needed to use feedback to improve performance across lists. It may be that the increased executive demands in the present task made it more vulnerable to effects of stereotype threat. It is also possible that the focus of the task on obtaining as many points as possible may have oriented participants toward gains and created a regulatory mismatch in the Stereotype Threat group. It is, therefore, possible that this mismatch reduced metamemory performance in this group. Future research could address this possibility by manipulating reward structure in order to compare effects of threat across gain-based and loss-based versions of the task (e.g., Barber and Mather, 2013a).

Previous research on stereotype threat has indicated that its effects may be more pronounced in older adults on the younger end of the age spectrum (Hess et al., 2009; Eich et al., 2014). For this reason, we recruited participants from a wide age range and tested for the effects of age on task performance. We found that both recall and betting declined as age increased, and there was no relationship between age and calibration or gamma. These findings are consistent with the idea that metamemory is intact in aging, even if memory abilities continue to decline. Contrary to our hypothesis, we did not find evidence of larger stereotype threat effects in older adults on the younger end of the age spectrum. It is possible that this was due to characteristics of our sample; for example, older adults on the older end of the age spectrum may remain susceptible to stereotype threat if they have demands on them in the real world that require a high level of cognitive performance.

A limitation of the present study is that the sample size was relatively small. While we obtained statistically significant differences between the conditions in terms of overall score, gamma coefficient, and number of bad bets in later lists, we obtained a number of marginally significant effects of condition, such as on the number of points lost, calibration score, and the effect of condition on performance across lists. This suggests a need for replication in a larger sample to determine whether these effects are reliable.

Taken together, our study provides preliminary evidence suggesting that age-related stereotype threat impairs older adults’ ability to prioritize high-value information. The pattern of results we observed suggests that metacognition, a process that is generally found to be intact in normal aging, may be susceptible to the negative impact of stereotype threat. Given the overlap between metacognitive abilities and executive control, it may be the case that both are burdened when stereotype threat is introduced to older adults while performing a memory task. In daily life, it is possible that the effect of stereotype threat on metacognition exacerbates its deleterious effect on memory. For example, older adults under stereotype threat may have reduced awareness of when they need to use external aids such as notes when confronted with information of varying significance. Our findings point to the importance of studying age-related stereotypes and how they may impact the day-to-day lives of older adults. An important goal for future research will be to find ways to lessen the effects of age-related stereotype threat in real-world situations. Interventions aimed at reducing the effects of stereotype threat can be implemented at the level of the environment (e.g., Purdie-Vaughns et al., 2008) or the individual (e.g., Taylor and Walton, 2011); a potential next step for this line of research would be determining what interventions are most effective at reducing the stereotype threat effects we observed.

## Data Availability Statement

The raw data supporting the conclusions of this article will be made available by the authors, without undue reservation.

## Ethics Statement

The studies involving human participants were reviewed and approved by Institutional Review Board of the University of California, Los Angeles. The patients/participants provided their written informed consent to participate in this study.

## Author Contributions

NF, AC, and BK contributed to conception and design of the study. NF oversaw data acquisition and wrote the first draft of the manuscript. TP assisted with data analysis and manuscript preparation. CL assisted with data acquisition and data analysis. All authors contributed to manuscript revision and read and approved the submitted version.

### Conflict of Interest

The authors declare that the research was conducted in the absence of any commercial or financial relationships that could be construed as a potential conflict of interest.

## Appendix

Neutral paragraph:

Scientific research has shown that our brains change as we get older. These changes do not prevent older adults from living active and independent lives. You can use many techniques that help with everyday situations that require the use of cognitive skills, for example, using a notepad to write down important things. Next, you will be presented with a cognitive task in which older adults (50 years of age and above) perform as well as younger adults. Please, do your best!

Please write your study ID number:_________

Stereotype Threat paragraph:

Scientific research has shown that brain areas linked to learning and memory deteriorate as we get older. As a result, memory function may be affected with age. One technique you can adopt to help with everyday situations that require the use of memory is asking younger family members or friends for help. Next, you will be presented with a memory task that older adults (50 years of age and above) find difficult. Please try your best.

Please write your age:__________
